# Validation and Application of an HPLC–UV Method for the Quantification of 3‐*O*‐Methylquercetin in Nasal Mucosa

**DOI:** 10.1002/elps.70093

**Published:** 2026-04-14

**Authors:** Jeniffer Viviana Ramirez Hernández, Laura da Silva Machado, Flávia Nathiely Silveira Fachel, Sara Elis Bianchi, Elizandra Braganhol, Sheila Porto de Matos, Valquíria Linck Bassani

**Affiliations:** ^1^ Programa de Pós‐Graduação em Ciências Farmacêuticas Universidade Federal do Rio Grande do Sul Porto Alegre Brazil; ^2^ Programa de Pós‐Graduação em Biociências Universidade de Ciências da Saúde de Porto Alegre Porto Alegre Brazil

**Keywords:** bioanalytical method validation, HPLC, 3*‐O‐*methylquercetin, nasal mucosa

## Abstract

3*‐O‐*methylquercetin (3*O*MQ) is a methylated flavonoid with relevant biological activity whose reliable quantification in complex biological matrices remains analytically challenging due to its low aqueous solubility and matrix‐related interferences. In the present study, a previously reported HPLC‐UV method was adapted and revalidated for quantifying 3*O*MQ in porcine nasal mucosa for use in in vitro permeation and retention studies. Chromatographic separation was achieved under isocratic conditions using a phenyl stationary phase, providing adequate selectivity and reproducibility in the new biological matrix. The method demonstrated excellent linearity over the concentration range of 1–25 µg/mL (*r* ^2^ > 0.99). Accuracy and precision met international regulatory acceptance criteria, with relative standard deviations below 5% for both intra‐day and inter‐day evaluations. It also demonstrated excellent robustness under deliberate variations in flow rate, temperature, and detection wavelength (RSD < 2.00%). Matrix effect (86.94%–110.28%) and recovery (85.21%–106.47%) results confirmed the reproducibility and the efficiency of the method. The applicability of the revalidated method was demonstrated through in vitro release, permeation, and retention experiments using Franz diffusion cells and porcine nasal mucosa. These findings demonstrate that the method is precise, sensitive, and robust, making it suitable for nasal permeation and retention studies of 3*O*MQ. Moreover, this analytical approach represents a reliable tool for in vitro and *ex vivo* pharmaceutical research, supporting the development and evaluation of nasal drug delivery systems for poorly soluble bioactive compounds, particularly flavonoid aglycones.

## Graphical abstract

This study improved an HPLC–UV method for quantifying 3‐*O*‐methylquercetin in porcine nasal mucosa following ICH M10 and ANVISA (Brazil) guidelines, assessing selectivity, linearity, accuracy, precision, and robustness. The method was applied for the first time to evaluate release, permeation, and retention profiles of a nasal formulation.

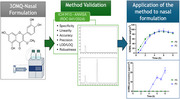

## 1 Introduction

The reliable quantification of bioactive flavonoids in complex biological matrices remains a significant analytical challenge, particularly for compounds with low aqueous solubility and strong interactions with endogenous matrix components. In this context, the validation of fit‐for‐purpose bioanalytical methods is essential to ensure reliable and reproducible quantification, as well as to confirm the suitability of the analytical approach for reliable analyte identification and quantification in the presence of matrix‐related interferences, thereby minimizing systematic errors in in vitro and ex vivo studies. [1]. Furthermore, the assessment of critical parameters, such as the limit of quantification, robustness, accuracy, precision, and matrix effect, is essential for the proper interpretation of bioanalytical results in complex systems [2].

**FIGURE 1:**
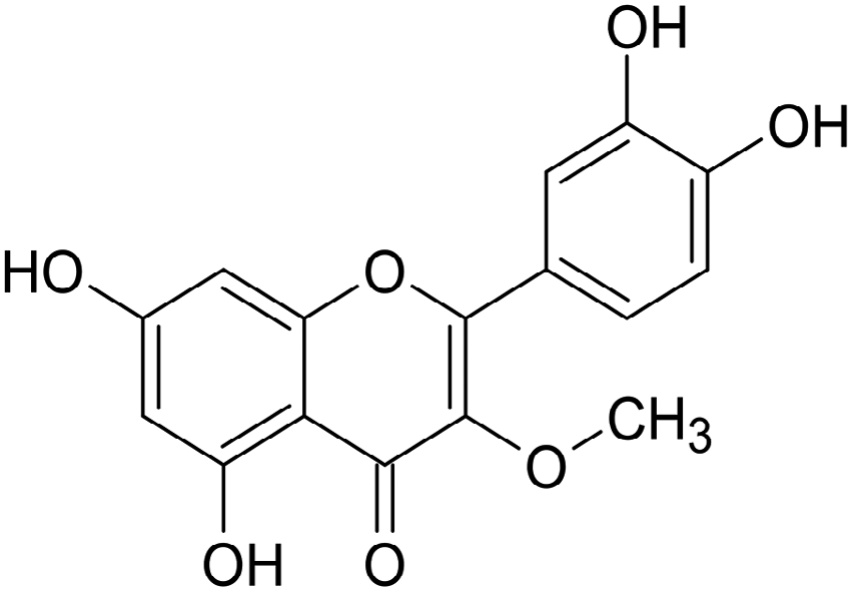
Chemical structure of 3*‐O‐*methylquercetin (5,7,3′,4′‐tetrahydroxy‐3‐methoxyflavone; 3*O*MQ).

3*‐O‐*methylquercetin (3*O*MQ) is a methylated flavonoid structurally derived from quercetin and has been identified in several plant species, including *Nicotiana tabacum*, *Solanum rostratum*, and *Achyrocline satureioides* [3, 4, 5]. This compound has been associated with a broad spectrum of biological activities, notably antioxidant, anti‐inflammatory, antitumor, and neuroprotective, highlighting its potential therapeutic value for various pathological conditions [6, 7, 8, 9]. However, 3*O*MQ exhibits limited solubility in aqueous media due to its aglycone nature and the increased hydrophobicity resulting from methylation of the hydroxyl group. This characteristic limits its incorporation into hydrophilic formulations, its bioavailability, and, consequently, its therapeutic potential, despite its significant pharmacological properties [10]. The molecular structure of 3*O*MQ is shown in Figure 1.

Inclusion complexes of 3‐*O*‐methylquercetin (3*O*MQ) with cyclodextrins (CDs) have been investigated as an effective strategy to improve the apparent aqueous solubility, stability, and handling of this hydrophobic flavonoid, taking advantage of the characteristic CD architecture, which comprises a lipophilic internal cavity capable of hosting hydrophobic molecules and a hydrophilic outer surface that favors interaction with aqueous media [11]. In parallel, the incorporation of biocompatible polymers such as chitosan has been explored as a means to modulate drug diffusion and enhance interactions with biological membranes, particularly in mucosal systems [12]. In this context, previous studies by our research group demonstrated that the complexation of 3*O*MQ, present in a flavonoid‐rich fraction of *A. satureioides*, with β‐cyclodextrin (βCD) improved its physicochemical properties [13]. However, the practical application of natural βCD is limited by its low aqueous solubility and toxicity concerns associated with certain administration routes. Therefore, hydroxypropyl‐β‐cyclodextrin (HPβCD) was selected in the present study due to its high water solubility, favorable safety profile, and broad applicability in pharmaceutical systems, including nasal administration [11, 14, 15, 16]. The incorporation of the 3*O*MQ/HPβCD complex into a chitosan matrix thus provides a suitable experimental system to support analytical method evaluation in a biologically relevant and matrix‐challenging context.

From an analytical perspective, the quantification of 3*O*MQ has been previously addressed by Bidone et al. [4], who developed and validated a bioanalytical method for quantifying 3*O*MQ in porcine skin after application of *A. satureioides* extracts. Their study focused on a nanoemulsion containing a dry extract from the species designed for cutaneous delivery. The analytical method was intended to assess the in vitro performance of this formulation as a cutaneous drug delivery system [4]. However, to date, no bioanalytical method validation studies of 3*O*MQ in porcine nasal mucosa have been reported, as the applicability of the method reported by Bidone et al. [4] was restricted to skin tissue and to the experimental and regulatory context in place at the time of its development.

In addition, current regulatory expectations for bioanalytical method validation have evolved, with updated international and national guidelines emphasizing the need for validation strategies tailored to the intended application of the method. The implementation of the ICH M10 guideline and the recent ANVISA Resolution RDC n° 941/2024 reinforce the importance of systematically evaluating critical validation parameters when established methods are applied to new matrices or experimental purposes [17, 18].

Within this context, the main objective of the present study was to validate a bioanalytical method, based on the method reported by Bidone et al. [4], for quantifying 3*O*MQ in porcine nasal mucosa, using a pharmaceutical formulation for intranasal administration, composed of cyclodextrin as a solubilizing agent and chitosan to promote nasal retention. A specific validation approach was employed to ensure the method's suitability for in vitro and ex vivo permeation and retention studies. By extending the applicability of the validated method to a new biological matrix and regulatory framework, this work provides a robust and reliable bioanalytical tool for the quantification of 3*O*MQ in complex nasal mucosa matrices.

## 2 Materials and Methods

### 2.1 Chemicals and Reagents

The 3*O*MQ compound (>97% purity) was previously obtained by extraction from the inflorescences of *A. satureioides*. This raw material was provided by the Multidisciplinary Center for Chemical, Biological, and Agricultural Research at the State University of Campinas (CPQBA‐UNICAMP, São Paulo, Brazil). A reference specimen (No. 308) was deposited in the CPQBA/UNICAMP herbarium, and access to genetic heritage in Brazil was registered in the SisGen system (Sistema Nacional de Gestão do Patrimonio Genético e Conhecimento Associado) under No. A732800. Water (H_2_O) was purified using a Milli‐Q system (TGI Pure Water Systems, Brea, CA, USA). Methanol (MeOH), acetonitrile (ACN), and trifluoroacetic acid were of HPLC grade (Tedia, Fairfield, OH, USA). Sigma‐Aldrich Co. supplied 2‐hydroxypropyl‐β‐cyclodextrin (HPβCD) with DS ≈ 0.80 (São Paulo, Brazil), as well as low‐molecular‐weight chitosan.

### 2.2 Preparation of the Stock and Work Solutions

Stock solutions of 3*O*MQ were prepared by accurately weighing 10 mg of the compound into a 10 mL volumetric flask, dissolving it, and making up the volume with methanol. For simultaneous quantitative analysis, appropriate aliquots of the stock solution were transferred to prepare a standard mixture solution at 100 µg/mL (1 µg/mL = 1 mg/L). Subsequently, a series of standard solutions with final concentrations ranging from 1 to 25 µg/mL was prepared by diluting with methanol to establish a calibration curve. Working standard solutions were prepared to facilitate quantitative analysis and to evaluate various validation parameters. Both stock and working solutions were maintained refrigerated at approximately 4°C throughout the experimental procedures.

### 2.3 Preparation of the Biological Matrix

The porcine nasal mucosa was extracted with 30 mL of methanol, with the biological samples immersed in the solvent for at least 24 h at 4°C before selectivity and matrix effect testing. The extended extraction period was used to promote the efficient transfer of endogenous matrix components into the solvent, generating a representative biological matrix for subsequent bioanalytical validation experiments. The low temperature was maintained to minimize potential degradation of matrix constituents.

The extracts were filtered through a 0.45 µm PTFE syringe filter (Captiva), and the supernatants were stored at 4°C until further analysis. This extraction procedure produces a simplified matrix extract suitable for bioanalytical validation in in vitro and ex vivo permeation and retention studies while ensuring adequate matrix consistency for method evaluation.

### 2.4 Preparation of the Chitosan‐Based Formulation

The 3*O*MQ/HPβCD complex was prepared by an organoaqueous method using a mixture of ethanol:water (40:60) as solvent. A 0.1% (w/v) chitosan dispersion was then prepared in distilled water, and the complex was subsequently incorporated into the polymeric matrix [19, 20, 21].

### 2.5 Chromatographic System and Conditions

The liquid chromatography system used was a Shimadzu LC‐20AT, equipped with an LC‐20AT pump, a CBM‐20A system controller, an SIL‐20A autosampler, and an SPD‐20AV UV/VIS detector (at 362 nm). The column employed was a ZORBAX Eclipse XDB‐Phenyl (Agilent Technologies) measuring 150 × 4.6 mm i.d., 3.5 µm, protected by a C18 guard column (20 × 3.9 mm, 10 mm i.d.) (Waters, USA) [4]. The mobile phase consisted of acetonitrile, methanol, and water containing 0.1% (v/v) trifluoroacetic acid (10:44:46, v/v/v). The injection volume was 20 µL, and elution was carried out under isocratic conditions at a flow rate of 0.8 mL/min. The system temperature was maintained at 30 ± 1°C, and detection was performed at 362 nm. These conditions were based on those reported by Bidone et al. [4].

### 2.6 Method Validation

The validation experiment was conducted following applicable recommendations of the ICH M10 guideline on bioanalytical method validation (ICH M10—Bioanalytical Method Validation, European Medicines Agency, 2022) and the recommendations of the Brazilian Agência Nacional de Vigilância Sanitária (ANVISA), as defined in Resolution RDC n° 941/2024, using a fit‐for‐purpose approach for in vitro and ex vivo permeation and retention studies [17, 18].

#### 2.6.1 Selectivity and Specificity

The ability of an analytical method to differentiate and quantify the analyte in the presence of potentially interfering substances, either from the biological matrix or the blank formulation, was evaluated based on the retention time of 3*O*MQ. Spectra, peak areas, and retention times of the analyte were compared between samples of the biological matrix and the formulation. The method is considered selective and specific when no interferences are observed near the analyte's retention time.

#### 2.6.2 Calibration Curve and Linearity

A calibration curve was constructed using six concentration levels (1, 5, 10, 15, 20, and 25 µg/mL), covering a range from 1 to 25 µg/mL, with each concentration analyzed in quintuplicate over three different days. Peak areas obtained from the chromatograms were plotted against theoretical concentrations and analyzed by least‐squares linear regression to determine the slope, y‐intercept, and correlation coefficient. Calibration curves were constructed using unweighted linear regression, as this model provided adequate accuracy and precision across the validated concentration range, within the method's fit‐for‐purpose context.

#### 2.6.3 Limits of Detection and Lower Limit of Quantification

The limits of detection (LOD) and quantification (LOQ) were calculated from the parameters of the regression equation, in accordance with the guideline ICH M10 (2022). LOD and LOQ were estimated based on the calibration curve parameters. The lower limit of quantification (LLOQ), defined as the lowest concentration level of the calibration curve (1 µg/mL), was experimentally verified through accuracy and precision assessments and met the predefined acceptance criteria. Therefore, the LLOQ was considered the relevant sensitivity parameter for the intended in vitro and ex vivo applications of the method, in accordance with the acceptance criteria established by ANVISA and ICH M10 guidelines [17, 18].

#### 2.6.4 Precision and Accuracy

Precision was evaluated using six replicates at each concentration quality control (QC) level, namely the lower limit of quantification (LLOQ), low (LQC), medium (MQC), high (HQC), and upper limit of quantification (ULOQ), analyzed on the same day (intra‐day) and across three different days (inter‐day). Results were expressed as relative standard deviation (RSD). Similarly, accuracy was evaluated using six replicates on the same day and on consecutive days by comparing the measured concentrations with their corresponding nominal concentrations and expressing the results as a percentage of the nominal concentration. According to ANVISA and ICH M10, precision was considered acceptable when the relative standard deviation did not exceed 15% for QC levels and 20% for the LLOQ, while accuracy was considered acceptable when values were within 85%–115% of the nominal concentration and 80%–120% for LLOQ [17, 18].

#### 2.6.5 Robustness

The robustness was assessed by varying critical parameters such as temperature (±1°C), wavelength (±1 nm), and flow rate (±0.01 mL/min), with each condition analyzed in triplicate. During each experiment, all other parameters were kept constant at their optimal values. Robustness was evaluated by comparing accuracy, bias, and precision across the different conditions to assess the ability of the method to remain unaffected by minor changes in experimental parameters.

Robustness was assessed at the ULOQ level, as this concentration is representative of the working range of the method and represents the worst‐case scenario for detecting the impact of deliberate chromatographic variations within the appropriate context for the purpose of the study.

#### 2.6.6 Matrix Effect

The matrix effect, defined as the influence of sample components on the analyte signal, was evaluated by comparing peak areas in each study matrix spiked with standard solutions at LLOQ, MQC, and ULOQ levels, along with solutions prepared at the same concentrations without matrix. According to ICH M10 criteria, results are considered acceptable when accuracy is within ±15% of the nominal concentration, and precision is ≤15% [17]. The matrix effect was evaluated using extracted porcine nasal mucosa, as this represents the actual matrix subjected to chromatographic analysis within the appropriate context of the method.

#### 2.6.7 Recovery

The analyte solutions at LLOQ, MQC, and ULOQ levels were spiked into the specified matrices, namely porcine nasal mucosa extract and chitosan–cyclodextrin–based formulation, which were subsequently analyzed. Recovery was expressed as a percentage, calculated by comparing the results obtained in the presence of the matrix with those obtained at the same concentrations in the absence of the matrix. According to ANVISA Resolution RDC 941/2024 and ICH M10 (2022), recovery values within ≤15% are considered acceptable [17, 18]. Recovery values were considered acceptable based on their consistency and reproducibility across all concentration levels, rather than absolute recovery, in accordance with the purpose of validation.

### 2.7 Method Application

#### 2.7.1 In Vitro Release of 3OMQ From the Formulations

The in vitro release of 3*O*MQ at 1 mg/mL was evaluated from a 3*O*MQ solution in propylene glycol (PS) and from the 0.1% chitosan‐based formulation containing the 3*O*MQ/HPβCD complex (FC). The in vitro release study was performed following the method described by Fachel et al. [22], using a Franz diffusion cell with an approximate diffusion area of 1.80 cm^2^ and synthetic cellulose ester membranes with a pore size of 50 nm (Millipore). The receptor compartment was filled with 12 mL of a PBS/ethanol mixture (70:30, v/v), and the solution was maintained at 35 ± 1°C. A 500 µL sample was placed in the donor compartment, with the temperature maintained constant throughout the assay. To estimate the amount of 3*O*MQ released from the formulation over 8 h, 1.0 mL samples were withdrawn from the receptor fluid at 30, 60, 120, 180, 240, 300, 360, 420, and 480 min, and the same volume was replaced with fresh medium to maintain a constant receptor volume. All aliquots were filtered through a 0.45 µm PTFE syringe filter (Captiva) and analyzed by the previously validated HPLC‐UV method. The area under the curve (AUC) for PS and FC was calculated and compared using GraphPad Prism 5 software [22].

#### 2.7.2 Permeation/Retention of 3OMQ in Porcine Nasal Mucosa

The permeation and retention of 3*O*MQ in propylene glycol (PS) and the chitosan‐based formulation containing 3*O*MQ/HPβCD complex (FC) were evaluated using a Franz diffusion cell across porcine nasal mucosa membranes. The nasal mucosa was obtained from a local slaughterhouse (Harmonia, Brazil) and prepared according to the method described by Fachel et al. [22]. Before the experiment, the mucosa was immersed in phosphate‐buffered saline (PBS) at pH 7.4 for 30 min. Subsequently, the membrane was placed between the donor and receptor compartments, with the receptor compartment filled with 12 mL of a PBS/ethanol mixture (70:30). The receptor solution was maintained at a controlled temperature of 35 ± 1°C. In the donor compartment, 500 µL of each sample was added, maintaining constant immersion conditions. Samples of 1.0 mL were withdrawn from the receptor fluid at hourly intervals, and the same volume was replaced with fresh medium to maintain a constant receptor volume. After 8 h, the mucosa was cleaned with a cotton swab, rinsed with Milli‐Q water to remove any excess, removed from the diffusion cell, and cut into small pieces. The retained 3*O*MQ was extracted from the mucosa using methanol in an ultrasonic bath for 30 min. All aliquots were subsequently filtered and analyzed by the previously validated HPLC‐UV method [22].

#### 2.7.3 Statistical Analysis

Data from in vitro release, permeation, and retention studies were obtained using six independent Franz diffusion cells (*n* = 6) per experimental condition and are presented as mean ± standard deviation (SD). Statistical analyses were performed using GraphPad Prism software 8.0.1.

Inferential statistical analysis was applied only when comparisons between formulations were required. In this case, retention data were analyzed by one‐way analysis of variance (ANOVA), followed by Tukey's post hoc test to identify statistically significant differences between groups. A *p*‐value < 0.05 was considered statistically significant.

## 3 Results and Discussion

### 3.1 Validation of the 3*O*MQ Analytical Method

The analytical method for quantifying 3*O*MQ was validated to evaluate the release, permeation, and retention of the formulation intended for nasal application. The procedure was carried out in accordance with ANVISA Resolution RDC No. 941/2024 and the international guideline ICH M10 (2022) on bioanalytical method validation [17, 18]. Key parameters assessed included selectivity, specificity, matrix effect, recovery, response function (calibration curve within the LLOQ–ULOQ range), accuracy, precision, and robustness.

#### 3.1.1 Selectivity and Specificity

**FIGURE 2:**
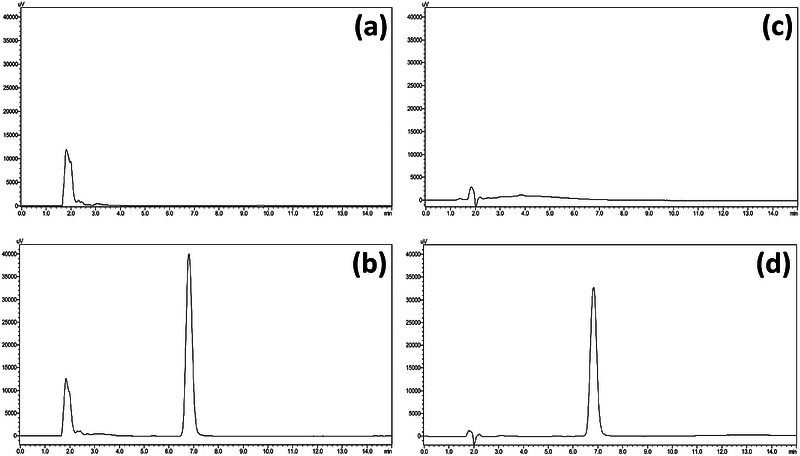
Liquid chromatography profile at 362 nm: (a) Chromatogram of porcine nasal mucosa extract. (b) Chromatogram of the biological matrix with 3*O*MQ at 10 µg/mL. (c) Chromatogram of the blank formulation. (d) Chromatogram of the 0.1% chitosan formulation with 3*O*MQ at 10 µg/mL. Analytical conditions: Column ZORBAX Eclipse XDB‐Phenyl, 150 × 4.6 mm, 3.5 µm; ACN:MeOH:H_2_O + 0.1 % TFA, 10:44:46 v/v/v; isocratic, 0.8 mL/min; 20 µL; 30°C.

The selectivity of the method was confirmed by the absence of interference from matrix components at the retention times of the analyte peak. This parameter was assessed by comparing chromatograms of the formulation and biological matrix with those of samples containing 3*O*MQ, confirming the high selectivity of the method (Figure 2). Furthermore, the method proved to be specific for this flavonoid, as no interferences were observed. This may be attributed to the lack of absorbance from the formulation and porcine nasal mucosa extract at the set wavelength, which shows only a weak signal around 2 min, without affecting the retention time or peak area of 3*O*MQ, consistent with findings reported by Bidone et al. [4].

In addition, system suitability was verified before analysis by assessing retention time repeatability and peak area precision, with relative standard deviations consistently below 2%. Adequate chromatographic separation was confirmed by the absence of interfering peaks near the retention time of 3*O*MQ in blank matrices and formulations.

#### 3.1.2 Linearity Curve and Limits of Detection and Quantification

The linearity of the method was assessed using linear regression of the calibration curve (Table 1). The determination coefficient (*r* ^2^) was calculated over a concentration range of 1 to 25 µg/mL, achieving values above 0.99, indicating a strong correlation. The slope and Y‐intercept were used to calculate the limits of detection (LOD) and quantification (LOQ), which were 0.346 and 1.049 µg/mL, respectively. The calibration curves demonstrated adequate linearity across the validated concentration range when fitted using unweighted linear regression, with residuals evenly distributed around zero and no systematic deviation observed at the lower concentration levels.

**TABLE 1:** Linearity curve of the HPLC analysis of 3*O*MQ.

Parameters	Results
Equation	*Y* = 129 165*x* – 20 833
*r* ^2^	0.9975
LOD (µg/mL)	0.346
LLOQ (µg/mL)	1.0
LOQ (µg/mL)	1.049
Intercept (confidence interval)	(−48 011.9; 6346.8)

#### 3.1.3 Sensitivity

The LLOQ is a critical parameter for validation, as it enables the quantification of low drug concentrations in biological matrices, as in the present study [23]. The sensitivity of the method was assessed at the LLOQ based on accuracy and precision results. Accuracy deviations were below 104%, and relative standard deviations below 5% for both intra‐day and inter‐day measurements. These values are well within the acceptance criteria established by ANVISA Resolution RDC No. 941/2024 and ICH M10 (2022) [17, 18], confirming the adequate sensitivity of the method.

#### 3.1.4 Precision and Accuracy

The precision and accuracy (Table 2) were evaluated at four QC levels: 1 µg/mL (LLOQ), 10 µg/mL (LQC), 15 µg/mL (MQC), and 25 µg/mL (ULQC), through five replicates, performed both on the same day (intra‐day) and over three different days (inter‐day). Intra‐day and inter‐day precision were ≤0.39% and ≤5.17%, respectively. Accuracy ranged from 98.73% to 103.31% for intra‐day determinations and from 101.93% to 103.61% for inter‐day determinations. These values fall within the criteria established by ANVISA and ICH for bioanalytical method validation, confirming that the employed method is suitable for the identification and quantification of compounds in porcine nasal mucosa samples with adequate precision and accuracy.

**TABLE 2:** Precision and accuracy data of the method.

3*O*MQ level (µg/mL)	Inter‐day^a^		Intra‐day^a^	
	Precision (RSD)	Accuracy (%)	Precision (RSD)	Accuracy (%)
1.0	4.79	103.61	3.74	101.99
10.0	4.88	101.99	0.85	98.73
15.0	5.17	101.93	0.39	98.27
25.0	3.24	103.56	1.19	103.31

^a^Precision was expressed as the relative standard deviation (RSD) of replicate measurements, and accuracy was expressed as a percentage of the nominal concentration.

#### 3.1.5 Robustness

During the robustness study, small deliberate variations were made in key analytical parameters to evaluate the robustness of the method and its ability to maintain consistent analytical performance under minor variations in method conditions. A single 3*O*MQ concentration of 25.0 µg/mL was used, and three different eluent flow rates (±0.01 mL/min) were tested, yielding an RSD of 1.79%. This demonstrated the robustness of the method against minor flow variations. Similarly, slight adjustments to the column temperature and detection wavelength resulted in RSDs of 1.47% and 1.36%, respectively, confirming that the method performance remained unaffected by these variations. These robustness studies demonstrate that the method can tolerate minor deliberate variations in the evaluated parameters while maintaining reliable results, with relative standard deviations remaining well within the acceptable range (RSD ≤ 3%) as recommended by ANVISA Resolution RDC No. 941/2024 and ICH M10 (2022) [17, 18]. The results are presented in Table 3.

**TABLE 3:** Robustness results of 3*O*MQ.

Changes in parameters		3*O*MQ level (µg/mL)	Mean peak area	SD	RSD^a^
Flow rate	±0.01 (mL/min)	25	3 301 684	59 103.42	1.79
Column temperature	±1(°C)		3 339 636	49 020.13	1.47
Detection wavelength	±1 (nm)		3 320 575	45 111.93	1.36

^a^RSD, relative standard deviation.

#### 3.1.6 Matrix Effect and Recovery

The normalized matrix factor percentage is within acceptable limits (the concentrations measured at all three levels are within 15% of the nominal value). These values were within the range of 86.94% to 110.28%. Although most concentrations showed slightly lower responses than the nominal values, this tendency was within the expected method variability and does not reflect a real decrease in the analytical response. In contrast, a slight increase in response was observed at the LLOQ level in the formulation, which also remained within acceptable limits. Additionally, the relative standard deviation (RSD) of the matrix factor in the two matrices ranged from 1.77% to 5.07%, as shown in Table 4.

**TABLE 4:** Matrix effect and extraction recovery for 3*O*MQ.

Matrix	3*O*MQ level (µg/mL)	Matrix effect		Recovery	
		Mean%	RSD	Mean%	RSD
Formulation	1	110.28	5.07	106.47	4.79
	10	92.90	4.43	91.20	5.17
	25	93.08	3.00	90.59	3.67
Porcine nasal mucosa extract	1	93.12	4.68	89.96	5.68
	10	87.80	1.77	86.27	5.27
	25	86.94	2.02	85.21	2.16

Analyte recovery after extraction ranged from 85.21% to 106.47%, with precision (RSD) between 2.16% and 5.27%, as detailed in Table 4. Recovery was consistent across QC levels, and precision well met the FDA Bioanalytical Method Validation guideline acceptance criterion of ≤15%. These results indicate that the analytical method provides consistent and reproducible responses across the evaluated matrices, meeting the applicable validation criteria established in current regulatory guidelines.

The results indicate that a method based on the approach proposed by Bidone et al. [4] can also be applied to other types of biological samples, such as porcine nasal mucosa [4]. The results are highly representative and indicate that the developed method allows rapid and accurate quantification of 3*O*MQ in the nasal mucosa. The low relative standard deviation (RSD% < 15%) supports this observation and aligns with previous studies on the presence of this flavonoid. This information is particularly relevant for permeation studies of nasal formulations containing the 3*O*MQ/HPβCD complex.

### 3.2 Method Application

#### 3.2.1 In Vitro Release of 3OMQ From the Formulation

To demonstrate the applicability of the validated HPLC‐UV method under experimental conditions involving drug release and diffusion, in vitro release experiments were performed through cellulose ester membranes using Franz diffusion cells. An amount of 500 µg of 3*O*MQ was applied to each diffusion cell.

**FIGURE 3:**
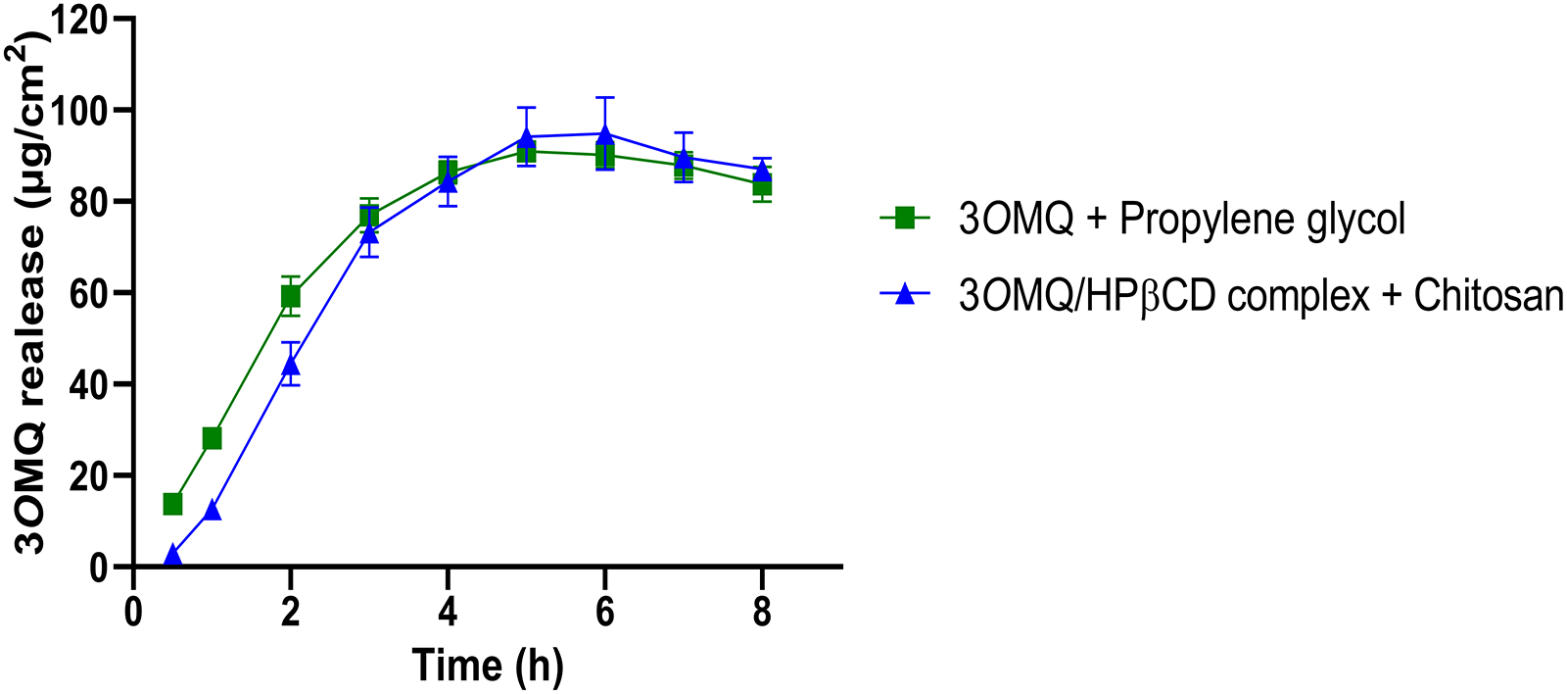
In vitro release profile through a synthetic cellulose membrane with a pore size of 50 nm (Millipore) of 3*O*MQ. Data represent mean ± SD (standard deviation).

Figure 3 shows the release of 3*O*MQ through synthetic cellulose ester membranes from the 3*O*MQ dispersion in propylene glycol (PS) and the chitosan‐based formulation (FC). After 8 h, the release reached 90.9 µg/cm^2^ for PS and 94.9 µg/cm^2^ for FC. Both systems produced comparable release profiles, enabling consistent quantification of 3*O*MQ throughout the experimental time course and demonstrating the suitability of the method for monitoring analyte concentrations under time‐dependent experimental conditions.

#### 3.2.2 Permeation and Retention of 3OMQ From the Formulation

Additionally, ex vivo permeation and retention experiments of 3*O*MQ from PS and FC across porcine nasal mucosa were conducted to further evaluate the performance of the validated method in a biological tissue matrix, with the same dose applied to enable comparison between the formulation profiles and a reference 3*O*MQ solution. This model was chosen to evaluate the intranasal permeation/retention potential of the drug in a matrix with morphological similarities to human nasal mucosa, while considering ethical aspects [24, 25]. To date, the permeation and retention of 3*O*MQ across the nasal mucosa using animal models have not been previously reported.

**FIGURE 4:**
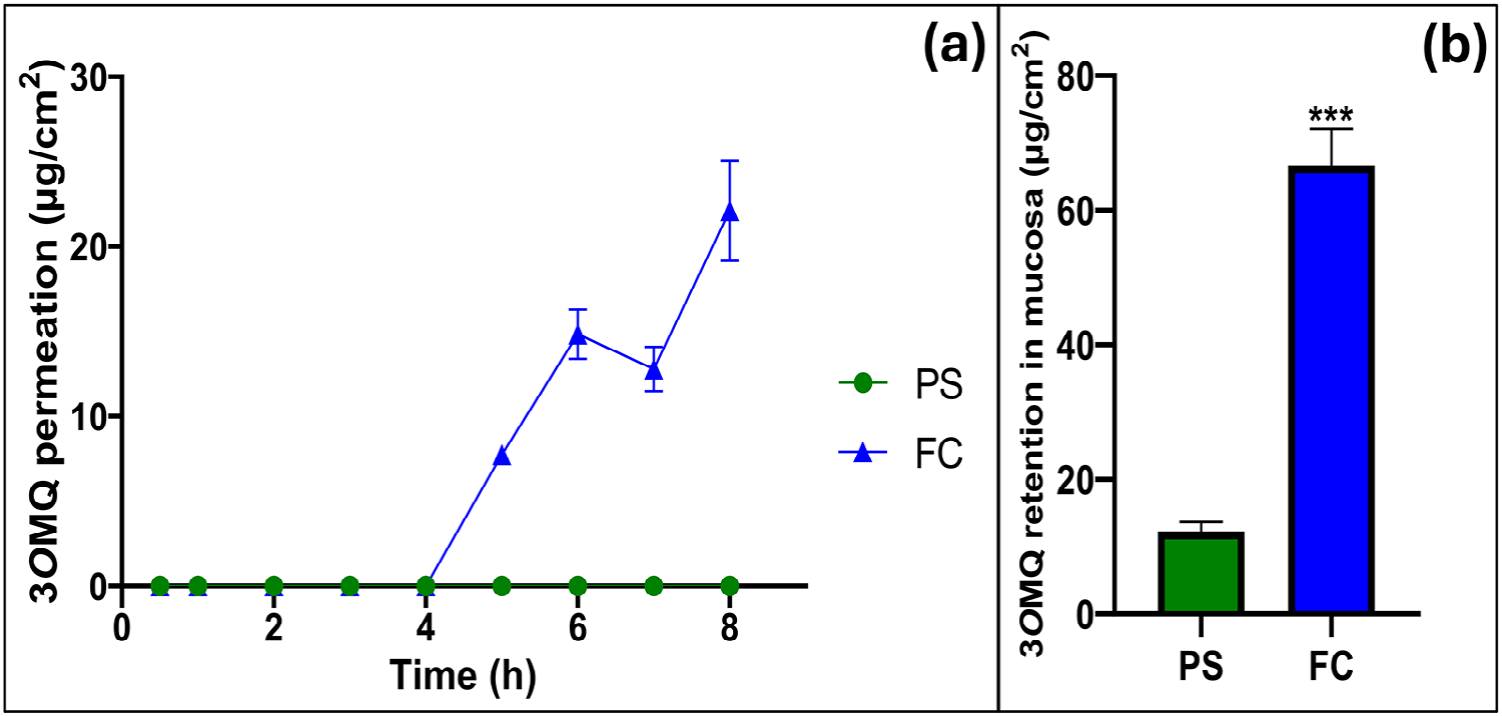
(a) Permeation and (b) retention profiles of 3*O*MQ through porcine nasal mucosa of (a). PS: 3*O*MQ in propylene glycol; FC: chitosan‐based formulation containing 3*O*MQ/HPβCD complex. Data are expressed as mean ± SD (standard deviation) (*n* = 6). Statistical analysis for retention data was performed using one‐way analysis of variance (ANOVA) followed by Tukey's post hoc test. ***p < 0.001 compared with PS.

The permeation profile of 3*O*MQ across porcine nasal mucosa for PS and FC is shown in Figure 4a. After 8 h of kinetic study, only the chitosan‐based formulation enabled analyte permeation, reaching approximately 22.12 µg/cm^2^. A summary of permeation and retention values obtained after 8 h, including the corresponding RSD%, is presented in Table 5. In contrast, permeation from the propylene glycol control solution remained below the LLOQ throughout the experiment, despite its previously observed faster and sustained release profile and the well‐documented permeability‐enhancing properties of propylene glycol [26]. Although the absolute permeation values were modest, they clearly demonstrated a differential permeation behavior between the formulation and the reference solution, with the chitosan‐based system enabling measurable permeation of 3*O*MQ across the nasal mucosa.

**TABLE 5:** Summary of permeation and retention through porcine nasal mucosa after 8 h.

Method application	Formulation	Mean ± SD (RSD)
Permeation (µg/cm^2^)	PS	<LLOQ
	FC	22.12 ± 2.97 (13.41)
Retention (µg/cm^2^)	PS	12.31 ± 1.42 (11.49)
	FC	66.68 ± 5.41 (8.11)

*Note*: Values are expressed as mean ± SD (*n* = 5).

Abbreviations: SD, standard deviation; RSD, relative standard deviation; LLOQ, lower limit of quantification; PS, 3*O*MQ dispersion in propylene glycol; FC, chitosan‐based formulation.

The observed permeation profiles primarily served to confirm the capability of the validated method to reliably quantify low analyte levels in porcine nasal mucosa under ex vivo experimental conditions. Although a detailed mechanistic interpretation of permeation was beyond the scope of the present analytical study, it is worth noting that β‐cyclodextrin derivatives such as HPβCD have been reported to enhance the aqueous solubility of hydrophobic compounds, favoring their availability for permeation. In addition, the presence of positively charged chitosan in the slightly acidic nasal environment has been described as a factor that may further facilitate analyte diffusion across mucosal barriers [27, 28].

Regarding the amount of 3*O*MQ retained in porcine nasal mucosa after 8 h, FC showed significantly higher retention (*p* < 0.001) compared with the propylene glycol solution, reaching 66.7 µg/cm^2^, as illustrated in Figure 4b. PS showed significantly lower retention of 12.3 µg/cm^2^, suggesting that the chitosan‐based system favored greater drug interaction with the nasal mucosa. These findings highlight the measurable permeation and the markedly higher retention of 3*O*MQ within the nasal mucosa are likely due to interactions between cationic chitosan particles and the negatively charged sialic acid residues in mucus and cell membranes. These electrostatic interactions help maintain the flavonoid at the nasal mucosa site and extend its local residence time [27, 28].

It should be emphasized that these experiments were not intended to fully characterize formulation performance or to establish a complete mass balance, but rather to demonstrate the applicability of the revalidated bioanalytical method for the reliable quantification of 3*O*MQ in complex biological matrices under conditions relevant to in vitro and ex vivo permeation and retention studies.

## 4 Conclusion

In this study, a bioanalytical HPLC–UV method originally reported by Bidone et al. [4] for porcine skin formulation was successfully revalidated for the quantification of 3*O*MQ in porcine nasal mucosa. The validation strategy followed a purposeful approach, addressing current regulatory expectations and demonstrating adequate selectivity, linearity, sensitivity, precision, accuracy, robustness, and control of matrix effects in a complex biological matrix.

The applicability of the revalidated method was further demonstrated through in vitro and ex vivo experiments designed to challenge its performance under conditions relevant to permeation and retention studies, confirming its suitability for reliable quantification of low analyte levels over the experimental time course.

In this context, the present study provides a robust and reproducible bioanalytical tool for the quantification of 3*O*MQ in nasal mucosa, supporting future in vitro and ex vivo investigations by applying an established analytical method to a new biological matrix relevant for nasal drug delivery.

## Author Contributions

Jeniffer Viviana Ramirez Hernández: conceptualization; writing – original draft. Laura da Silva Machado: Investigation; methodology. Sara Elis Bianchi: Project administration; writing – review and editing. Elizandra Braganhol: conceptualization and writing – review and editing. Flavia Nathiely Silveira Fachel: Project administration, writing – review and editing. Sheila Porto de Matos: Project administration; writing – review and editing. Valquíria Linck Bassani: conceptualization; funding acquisition; project administration; writing – review and editing.

## Conflicts of Interest

The authors declare no conflicts of interest.

## Data Availability

The data that support the findings of this study are available from the corresponding author upon reasonable request.
